# Molecular Evidence for a Functional Ecdysone Signaling System in *Brugia malayi*

**DOI:** 10.1371/journal.pntd.0000625

**Published:** 2010-03-09

**Authors:** George Tzertzinis, Ana L. Egaña, Subba Reddy Palli, Marc Robinson-Rechavi, Chris R. Gissendanner, Canhui Liu, Thomas R. Unnasch, Claude V. Maina

**Affiliations:** 1 New England Biolabs, Ipswich, Massachusetts, United States of America; 2 Department of Entomology, University of Kentucky, Lexington, Kentucky, United States of America; 3 Department of Ecology and Evolution, University of Lausanne, Lausanne, Switzerland; 4 Global Health Infectious Disease Research Program, College of Public Health, University of South Florida, Tampa, Florida, United States of America; University of Pittsburgh, United States of America

## Abstract

**Background:**

Filarial nematodes, including *Brugia malayi*, the causative agent of lymphatic filariasis, undergo molting in both arthropod and mammalian hosts to complete their life cycles. An understanding of how these parasites cross developmental checkpoints may reveal potential targets for intervention. Pharmacological evidence suggests that ecdysteroids play a role in parasitic nematode molting and fertility although their specific function remains unknown. In insects, ecdysone triggers molting through the activation of the ecdysone receptor: a heterodimer of EcR (ecdysone receptor) and USP (Ultraspiracle).

**Methods and Findings:**

We report the cloning and characterization of a *B. malayi* EcR homologue (*Bma-EcR*). Bma-EcR dimerizes with insect and nematode USP/RXRs and binds to DNA encoding a canonical ecdysone response element (EcRE). In support of the existence of an active ecdysone receptor in *Brugia* we also cloned a *Brugia rxr* (retinoid X receptor) homolog (*Bma-RXR*) and demonstrate that Bma-EcR and Bma-RXR interact to form an active heterodimer using a mammalian two-hybrid activation assay. The Bma-EcR ligand-binding domain (LBD) exhibits ligand-dependent transactivation via a GAL4 fusion protein combined with a chimeric RXR in mammalian cells treated with Ponasterone-A or a synthetic ecdysone agonist. Furthermore, we demonstrate specific up-regulation of reporter gene activity in transgenic *B. malayi* embryos transfected with a luciferase construct controlled by an EcRE engineered in a *B. malayi* promoter, in the presence of 20-hydroxy-ecdysone.

**Conclusions:**

Our study identifies and characterizes the two components (*Bma-EcR* and *Bma-RXR*) necessary for constituting a functional ecdysteroid receptor in *B. malayi*. Importantly, the ligand binding domain of BmaEcR is shown to be capable of responding to ecdysteroid ligands, and conversely, ecdysteroids can activate transcription of genes downstream of an EcRE in live *B. malayi* embryos. These results together confirm that an ecdysone signaling system operates in *B. malayi* and strongly suggest that Bma-EcR plays a central role in it. Furthermore, our study proposes that existing compounds targeting the insect ecdysone signaling pathway should be considered as potential pharmacological agents against filarial parasites.

## Author Summary

Filarial parasites such as *Brugia malayi* and *Onchocerca volvulus* are the causative agents of the tropical diseases lymphatic filariasis and onchocerciasis, which infect 150 million people, mainly in Africa and Southeast Asia. Filarial nematodes have a complex life cycle that involves transmission and development within both mammalian and insect hosts. The successful completion of the life cycle includes four molts, two of which are triggered upon transmission from one host to the other, human and mosquito, respectively. Elucidation of the molecular mechanisms involved in the molting processes in filarial nematodes may yield a new set of targets for drug intervention. In insects and other arthropods molting transitions are regulated by the steroid hormone ecdysone that interacts with a specialized hormone receptor composed of two different proteins belonging to the family of nuclear receptors. We have cloned from *B. malayi* two members of the nuclear receptor family that show many sequence and biochemical properties consistent with the ecdysone receptor of insects. This finding represents the first report of a functional ecdysone receptor homolog in nematodes. We have also established a transgenic hormone induction assay in *B. malayi* that can be used to discover ecdysone responsive genes and potentially lead to screening assays for active compounds for pharmaceutical development.

## Introduction

Human filarial parasitic nematodes are responsible for two chronic severely debilitating tropical diseases: lymphatic filariasis and onchocerciasis. The global efforts in the treatment and control of the spread of infection for both parasites so far have resulted in limited success. Also, the widespread use of the few available specific drugs for fighting these diseases raises the possibility of the development of drug resistance [1]. With 140 million cases of infection worldwide, and over a billion people at risk of contracting these debilitating diseases [2], the development of a wide range of therapeutic interventions and treatment options is urgent.

Filarial parasites spend portions of their life cycle in obligate mammalian and insect hosts. The completion of a successful life cycle requires the passage of the developing nematode through four molts, two in the mammalian host and two in the arthropod host. The transmission of the parasite from one host to the other initiates a rapid molt, indicating that the developmental cues that trigger molting are closely tied to the integration of the parasitic larva into a new host environment. Inhibition of molting would result in the arrest of the life cycle in either the mammalian or insect host and the prevention of both pathology and/or the infective cycle. Thus, the study of the molting process in filarial nematodes could point to specific targets for drug development.

Molting in ecdysozoans [3] has been best characterized in insects. 20-hydroxyecdysone (20E) acts as the temporal signal to initiate molting, regulates embryogenesis, and coordinates tissue-specific morphogenetic changes in insects [4]–[6]. Ecdysone signaling is regulated by the activity of a heterodimeric receptor composed of two nuclear receptor proteins EcR and USP, although the hormone binding function resides only within EcR [7]–[10]. After ligand binding, EcR/USP activates a cascade of gene expression whose end result is the execution of molting [11].

Three alternatively spliced mRNA isoforms of *EcR* have been identified in *Drosophila* [12]. Mutations in these different *EcR mRNA* isoforms result in a range of phenotypes that includes lethality at the embryonic, larval and pupal stages, disruption of salivary gland degeneration [13], aberrant neuronal remodeling during metamorphosis [14], and changes in female fecundity and vitellogenesis [15].

EcR and USP, as well as a number of the proteins involved in the ecdysone-signaling cascade, are members of the nuclear receptor (NR) superfamily [10],[16]–[18]. NRs are characterized by significant amino acid sequence similarities in two key functional domains: the DNA binding domain (DBD), which directs the sequence-specific DNA-binding of the receptor, and the ligand binding domain (LBD), which mediates dimerization, ligand binding and transcriptional activation [19]–[20]. Some nuclear receptors have been shown to interact with a number of small molecule ligands such as metabolites and hormones, and these interactions are important for regulation of their activity. Other NRs are considered orphan receptors and are either not ligand-regulated or their cognate ligands have yet to be identified [19]. Homologs of the insect NRs that function downstream of EcR and USP have also been identified in filarial parasites (21, 22; Egaña, Gissendanner and Maina, unpublished results) as well as in the free-living nematode *C. elegans* [23]–[26]. Surprisingly, however, homologs of EcR or RXR/USP are apparently absent in the exceptionally large *C. elegans* NR family [23].

In filarial nematodes the molecular triggers of molting remain largely unknown. As in insects, a possible candidate for a signal that controls molting in *B. malayi*, the causative agent of lymphatic filariasis, is the steroid hormone 20E. Both free and conjugated ecdysteroids have been identified in the larvae of several parasitic nematodes including *Dirofilaria immitis* and *Onchocerca volvulus* [27]–[29]. In addition, ecdysteroids have been shown to exert biological effects on several nematodes. For example, in *Nematospiroides dubius* [30] and *Ascaris suum* [31] molting can be stimulated *in vitro* by low concentrations of ecdysteroids. Also, molting of third stage larvae of *D. immitis* can be stimulated with 20E and RH5849, an ecdysone agonist [32],[33]. The arrest at the pachytene stage of meiosis is abrogated when *D. immitis* ovaries are cultured *in vitro* with ecdysone and *B. pahangi* adult females can be stimulated to release microfilaria when cultured *in vitro* with ecdysone [34].

There appears to be a physiological connection between the filarial parasite and its arthropod host that may involve ecdysteroid signaling. Uptake of microfilaria (L1) by a feeding female mosquito at the time of a bloodmeal coincides with an increase in the production of mosquito ecdysteroids that results in the initiation of mosquito oocyte maturation [35]. Concurrent with this increase in ecdysteroid concentration in the mosquito host, larvae initiate a molt transition from L1 to L2 and later from L2 to L3, the infectious stage of the parasite. These observations suggest a potential role for ecdysone in the regulation of molting and other developmental processes in filarial nematodes.

We previously identified an *rxr* homolog in the dog filarial parasitic nematode *D. immitis* and demonstrated its ability to dimerize with an insect EcR and function in Schneider S2 cells [36]. We extend this work here with the identification and characterization of *EcR* and *rxr* homologs from *B. malayi*. Bma-EcR and Bma-RXR share some of the biochemical properties of insect EcR and RXR and show differences that appear to be nematode specific.

## Methods

### Parasites, RNA isolation and reverse transcription

*Brugia malayi* adult males, females or L1 larvae (TRS Labs, Athens, GA) were frozen in liquid nitrogen and ground with a pestle and mortar. Total RNA was purified from the pulverized tissue using RNAwiz (Ambion). RNA was quantified with a spectrophotometer and its quality assessed by gel electrophoresis. One µg of total RNA per isolation was reverse-transcribed using the ProtoScript first strand cDNA synthesis kit (New England Biolabs) following the manufacturer's protocol.

### Cloning of *Bma-EcR*, *Ov-RXR* and *Bma-RXR*

The genomic library from *B. malayi* in pBeloBAC vector gridded on Nylon filters (Filarial Genome Network, FGN, (http://www.nematodes.org/fgn/index.shtml) was screened using a cDNA fragment from a *D. immitis* EcR homolog (*Di-EcR*) (C. Shea, J. Richer and C. V. Maina, unpublished results) as a probe. Three positive BACs were identified and the individual corresponding bacterial clones were cultured. The inserts were confirmed to contain identical or overlapping sequences by restriction digestion analysis. One 11kb *Xba*I fragment identified by southern blot hybridization with the *Di-EcR* probe was subcloned into Litmus 28i and the insert was sequenced using GPS®-1 Genome Priming System (NEB) as directed by the manufacturer. PCR primers were designed to amplify *Bma-EcR* using sequence from the identified exons [37]. The primers used to amplify the full ORF were: 5′ – GGC GCT AGC ATG ACT ACA GCA ACA GTA ACA TAT CAT GAG TT – 3′ (Nco-MMT-5); 5′ – GGC CTC GAG CGA TTC TAT GGA TAG CCG GTT GAG GTT – 3′ (Xho-GYP-3). To determine the expression pattern and identify alternate isoforms of *Bma-EcR*, adult female, male, L1, L2, L3 cDNA libraries (FGN) were screened using the following primers: 5′ – GGG TAA TTC CTA CCA ACA GCT - 3′(GNS); 5′ – CAA GGG TCC AAT GAA TTC ACG AT – 3′ (GPL) corresponding to a fragment of the LBD from amino acids GNSYQQ to REFIGPL'. Additional sequence to extend the *Bma-EcR* isoforms identified was obtained by PCR, combining the latter two primers with the T3 and T7 promoter primers as their sequence is present in the library vector.

An *O. volvulus* L3 cDNA library (FGN) was screened by PCR using the following primers: 5′ – GAT CTT ATC TAT CTA TGC CGA GAA ,– 3′; 5′ – TAC TTT GAC ATT TGC GGT AAC GAC – 3′ corresponding to the amino acid sequence DLIYLCRE and RYRKCQSM of the conserved DNA binding domain of DiRXR-1 respectively. Additional *Ov-rxr* sequence was obtained by PCR using the same primers in combination with the T7 and T3 promoter primers. Candidate clones were identified by hybridization with a fragment of DiRXR-1 sequence. An amplified fragment from this library contained sequence corresponding to the *Ov-rxr* A/B and C domains.

Using BLAST, the *Di-rxr-1* sequence [36] was used to screen the *B. malayi* genome sequence available from The Institute for Genomic Research (TIGR) parasites database (http://blast.jcvi.org/er-blast/index.cgi?project=bma1). This analysis resulted in the identification of several exons encoding an 1189 bp fragment of open reading frame (ORF) that corresponded to a putative homolog of *rxr* in *Brugia* (*Bma-RXR*). Based on the genomic sequence we designed PCR primers and used them to amplify the expected *Bma-RXR* mRNA using a nested PCR approach. One µl of a reverse transcription reaction from female total RNA was used as the template for the first round of PCR carried out with primers 5′ – CGA TCT ATG CCC ATC AGA TTG-3′ (LCP) and 5′ – CAC AAT GCA AGC TAA GAG ATC G – 3′(RSL) at 46°C annealing temperature. Six percent of the first round PCR reaction was used as a template in a second round of PCR with primers 5′ – CGA TTT AAC TCC AAA TGG AAG TCG – 3′ (DLT) and 5 ′– AGC AAA GCG TTG AGT TTG TGT TGG – 3′ (PTQ) at 47°C annealing temperature. Using the sequence obtained, primers were designed to extend the 5′ of the coding sequence using a semi-nested PCR approach in combination with the 5′ splice leader SL1 primer. As above, two rounds of PCR were employed, in the first round using the SL1 primer (5′ – GGT TTA ATT ACC CAA GTT TGA G - 3′) and primer 5′ – GAT GCT CGA TCA CCG CAT ATT GCA CAA ATG - 3′ (CAI) at 68°C annealing temperature and for the second round the SL1 primer and primer 5′- TGG CAT ACA GTG TCA TAT TTG GTG TTG TGC - 3′ (STT) at 66°C annealing temperature. The 3′ coding sequence was obtained by 3′ RACE using the First Choice RLM-RACE kit (Ambion) with *Bma-RXR* primers 5′ – GGC TCT AAT GCT ACC ATC ATT TAA TGA A - 3′ (ALM), and 5′ – GAA GAT CAA GCT CGA TTA ATA AGA TTT GGA - 3′ (EDQ) following the manufacturer's protocol. For each amplified fragment several clones were sequenced. The positions of the primers used are indicated by short arrows over the corresponding amino acid sequence in the alignment Figures.

### Northern blot analyses

Ten µg of total RNA from adult male, adult female and microfilaria were used to carry out northern blot analyses using the NorthernMax-Gly kit (Ambion). The *Bma-EcR* and *Bma-RXR* probes were 1kb DNA fragments from the respective coding regions labeled using random priming with the NEBlot kit (NEB) and ^32^P-dATP (NEN-Dupont). The sizes of the hybridizing RNA species were estimated using an RNA ladder that was run adjacent to the samples as a reference.

### Phylogenetic analyses

Predicted amino acid sequences of cloned cDNAs were aligned with all nuclear receptor sequences from Swissprot and GenBank, using Muscle [38] with default options. A complete phylogeny of all the nuclear receptor super-family was built with PhyML [39]. Subsequently, phylogenies of the relevant sub-families were constructed with 1000 bootstrap replicates. Well aligned sites were selected with GBLOCKS [40], with relaxed options to allow a few gaps per column of the alignment. In each case PhyML was run with rate heterogeneity with 4 classes, parameter alpha estimated from the data, BIONJ starting tree. Support for nodes was estimated by Approximate Likelihood-Ratio Test (aLRT) [41].

### Protein-protein interaction by GST-pull-down assays

A cDNA fragment of *Bma-EcR* encoding aa 152–465 (upstream of the C-domain to the end of the predicted ORF) was amplified by PCR using primers 5′ – AGC TTC CAT GGC AGC TGA AGA AGG TCA ATC TAA TGG CGA CAG TGA GT – 3′ (536 to 557 of EF362469) and Xho-GYP-3 (See cloning of *Bma-EcR*). The fragment was cloned in frame with GST in the vector pGEX-KG [42]. The fusion protein was produced in *E. coli* BL21 by induction at 30°C with 0.1 mM IPTG and purified on Glutathione Sepharose beads (Pharmacia) as directed by the manufacturer. Recombinant *Di-rxr-1* and *Aausp* (gift from A. Raikhel, University of California Riverside) in pcDNA-3 (Invitrogen) were transcribed and translated *in vitro* in rabbit reticulocyte lysates using the TNT T7 coupled transcription-translation system (Promega) in the presence of ^35^S-Methionine (Amersham Biosciences) as recommended by the manufacturer. Glutathione resin beads loaded with 1 µg of GST:Bma-EcR fusion protein were incubated for 1 h at 4°C with 5 µl of rabbit reticulocyte lysate containing labeled proteins (Di-RXR-1 or AaUSP) in a total volume of 10 µl of binding buffer (20 mM Tris, 1 mM EDTA, 1 mM DTT, 10% glycerol, 150 mM sodium chloride, 0.5 mg/ml of BSA, complete protease inhibitor cocktail (Sigma). The beads were washed twice with binding buffer and three times with buffer without BSA, then incubated with 10 mM reduced glutathione to elute the proteins, and centrifuged. Supernatants were mixed with loading buffer and analyzed by SDS-PAGE. Signals were detected by autoradiography of the dried gels.

### DNA binding by electrophoretic mobility shift assays (EMSA)

The ecdysone response element (PAL-1) described by Hu et al. [43] was produced by annealing two synthetic oligonucleotides: 5′ – TTG GAC AAG GTC AGT GAC CTC CTT GTT CT – 3′ and its complement (with two overhanging Ts at each 3′ end). PAL-1 was labeled with ^32^P-dATP (NEN-Dupont) using Klenow polymerase (New England Biolabs) and purified by spin column G50 chromatography (Amersham Biosciences). A cDNA fragment containing the complete coding region of *Bma-EcRA* was cloned in pcDNA-3 (Invitrogen) using the *Nhe*I and *Xho*I restriction sites. Two additional constructs containing *Bma-EcRB* and *C* respectively were also cloned using the same strategy. The three *Bma-EcR* isoforms were transcribed and translated *in vitro* in rabbit reticulocyte lysates using the TNT T7 coupled transcription-translation system (Promega) following the manufacturer's protocol. The translation yield of each construct was assessed by labeling a portion of the reaction with ^35^S-Methionine and analyzing the products after gel electrophoresis and autoradiography. Binding reactions were performed at room temperature in 10mM Tris-HCl pH 7.5, 50 mM NaCl, 10 mM MgCl_2_, 0.5 mM DTT, 0.025 mM EDTA, 4% glycerol, 0.2 µg/µL poly dI-poly-dC, 0.13 µg/µL BSA, 0.05% NP40, with 13 fmol/µl labeled PAL-1 and 3.5 µL TNT reaction mixture containing the corresponding proteins (0.5 µLAaUSP and 1.5 or 2.5 µL Bma-EcR), in 15 µL total volume for 20 min before loading in a 6% native TBE gel (Invitrogen). Signals were detected by autoradiography of the dried gels.

### Constructs and transactivation assays in mammalian cells

To construct GAL4:Bma-EcR and VP16:Bma-RXR, the DEF domains of *Bma-EcR* and *Bma-RXR* were PCR amplified and cloned into pM and pVP16 vectors (EcR residues 259–565; RXR residues 191 to 464) respectively (Clontech). The construct VP16:Lm-HsRXREF (Chimera 9) has been previously described [44]. pFRLUC, encoding firefly luciferase under the control of the GAL4 response element (Stratagene Cloning Systems) was used as a reporter.

Fifty thousand NIH 3T3 cells per well in 12-well plates were transfected with 0.25 µg of receptor(s) and 1.0 µg of reporter constructs using 4 µl of SuperFect (Qiagen). After transfection, the cells were grown in medium containing ligands for 24–48 hours. A second reporter, Renilla luciferase (0.1 µg), expressed under a thymidine kinase constitutive promoter was cotransfected into cells and was used for normalization. The cells were harvested, lysed and the reporter activity was measured in an aliquot of lysate. Luciferase activity was measured using Dual-luciferaseTM reporter assay system from Promega Corporation (Madison, WI, USA). The results are reported as averages of normalized luciferase activity and the error bars correspond to the standard deviation from multiple assays. The ligands used were: RG-102240, a synthetic stable diacylhydrazine ecdysone agonist [N-(1,1-dimethylethyl)-N′-(2-ethyl-3-methoxybenzoyl)-3,5-dimethylbenzohydrazide] also known as GS-E or RSL1, (RheoGene, New England Biolabs) and Ponasterone-A (Invitrogen). The ligands were applied in DMSO at the indicated final concentrations and the final concentration of DMSO was maintained at <0.1%.

### Constructs and transactivation assays in *Brugia malayi* embryos

In order to construct an ecdysteroid response reporter for *Brugia malayi* the repeat domain of the *B. malayi* 12 kDa small ribosomal subunit gene promoter [45] (construct BmRPS12 (−641 to −1)/luc) was replaced (in both orientations) with the PAL-1 EcRE shown to be recognized by Bma-EcR *in vitro*. Previous studies have shown that the repeat acts as a transcriptional enhancer. Outward facing primers flanking the repeat domain containing synthetic *Spe*I sites at their 5′ ends were used in an inverse PCR reaction employing BmRPS12 (−641 to −1) as a template [46]. The resulting amplicons were purified using the QiaQuick PCR cleanup kit (Qiagen). The purified amplicons were digested with *Spe*I, gel purified, self-ligated and transformed into *E. coli*. The resulting construct was designated BmRPS12 -rep. A double stranded oligonucleotide consisting of five tandem repeats of the EcRE: ctag(GGACAAGGTCAGTGACCTCCTTGTTC) 5× with *Spe*I overhangs was then ligated into the *Spe*I site of BmRPS12 -rep. The insertions in the forward and reverse orientations were designated BmRPS12-EcRE and BmRPS12-EcRE-rev respectively.

Constructs were tested for promoter activity in transiently transfected *B. malayi* embryos essentially as previously described [47]. In brief, embryos were isolated from gravid female parasites and transfected with BmRPS12-EcRE (or BmRPS12-EcRE-rev) mixed with a constant amount of a transfection control, consisting of the BmHSP70 promoter fragment driving the expression of renilla luciferase (construct BmHSP70 (−659 to −1)/ren). Following a rest of five minutes, the transfected embryos were transferred to embryo culture media (RPMI tissue culture medium containing 25 mM HEPES, 20% fetal calf serum, 20 mM glucose, 24 mM sodium bicarbonate, 2.5 mg ml-1 amphotericin B, 10 units ml-1 penicillin, 10 units ml-1 streptomycin and 40 mg ml/L gentamycin), supplemented with 1 µM 20-OH ecdysone dissolved in 50% ethanol or solvent control. Transfected embryos were maintained in culture for 48 hours before being assayed for transgene activity. Firefly luciferase activity was normalized to renilla luciferase activity in each sample to control for variations in transfection efficiency. Firefly/renilla activity ratios for each sample were further normalized to the activity ratio from embryos transfected in parallel in each experiment with the parental construct BmRPS12 -rep. This permitted comparisons of data collected in experiments carried out on different days.

### Statistical analysis

Each construct was tested in two independent experiments, with each experiment containing triplicate transfections of each construct to be analyzed. The statistical significance of differences noted between the activity in the control and experimental transfections was determined using Dunnett's test, as previously described [47].

### Sequence accession numbers

The nucleotide sequences for Bma-EcR isoform A, Bma-EcR isoform C, Bma-RXR and Ovnhr-4 have been deposited in the GenBank database under GenBankAccession Numbers: EF362469, EF362470, EF362471, and EF362472.

## Results

### Bma-EcR cloning and genomic structure

**Figure 1:**
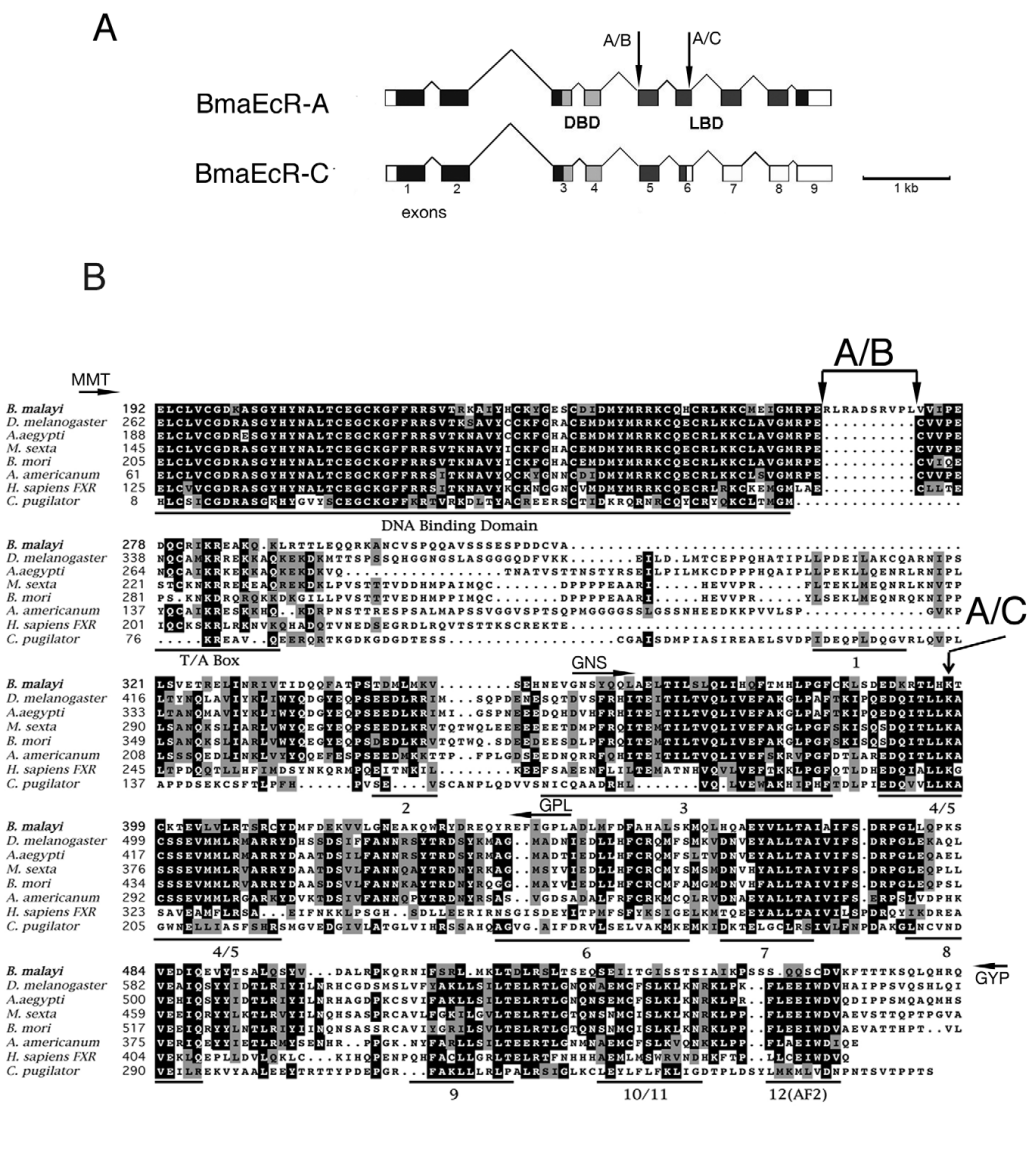
Bma-EcR genomic structure and protein sequence similarity to other EcRs. (A)Schematic representation of the genomic structure of *Bma-EcR A* and *-C* isoforms. The protein coding regions are shaded: DNA Binding Domain (DBD - light gray), Ligand Binding Domain (LBD - dark gray), other coding regions (black). Non-coding regions are white. Alternative splicing of exon 6 results in isoform C which is spliced 29 nucleotides downstream of the site used in isoform A (arrow A/C), changing the reading frame and prematurely terminating the LBD. An additional splice site in exon 5 would generate the putative isoform B (arrow A/B) described in the text and in B below. (B) Protein sequence alignment of the DBD and LBD of Bma-EcR with ecdysozoan EcRs and FXR. The DNA Binding Domain, T/A Box, LBD helices and AF2 region are underlined, and amino acid residue numbers are indicated. Residues that are found in a majority of the EcRs shown are shaded in black. Residues that are biochemically similar are shaded in gray. The position K397 where Bma-EcRC diverges from Bma-EcR A is indicated by an arrow labeled A/C. In Bma-EcR C, K397 is followed by the sequence DITLLRHV and a termination codon. The 10 amino acids that would be omitted in putative isoform B are marked by the two arrowheads and labeled (A/B). Position of PCR primers used for screening and cloning have been indicated by a small arrow labeled by the corresponding 3 amino acid residue name (see experimental procedures for primer names). Primers MMT and GYP cover regions outside the alignment.

A candidate *EcR* homolog was first identified from *D. immitis* using degenerate PCR primers based on insect EcRs (C. Shea, J. Richer and C.V. Maina, unpublished results). Using sequences from the *D. immitis EcR* homolog, genomic libraries from *B. malayi* available from the Filarial Genome Network (FGN) were screened. A strongly hybridizing BAC was identified and sequenced. This BAC contained a gene that encodes a protein with strong similarities to the *EcR* branch of nuclear receptors (see below). We designated this gene as *Bma*-EcR (to distinguish it from *Bombyx mori* EcR [37]). Using sequences corresponding to the predicted *Bma-EcR* exons, PCR primers were designed and used to screen larval and adult cDNA libraries. This library survey revealed *Bma-EcR* expression in L1, L3, and L4 larval stages, as well as in adult males and females (data not shown). In the microfilaria (L1) library, using primers from the putative ligand-binding domain (LBD) encoding region, two alternatively spliced mRNA isoforms of *Bma-EcR* (isoforms A and C) were identified (Fig. 1A). *Bma-EcRA* is the isoform containing the longest ORF (597 a.a.) with an intact LBD. *Bma-EcRC* contains exon 6 with a 29-nucleotide deletion that results in a reading frame shift that generates a premature stop codon and truncation of the LBD at helix 5 (Fig. 1). This is the result of an alternative splice site within exon 6. In addition to these confirmed isoforms, a splice site consensus sequence was identified within exon 5 at the end of the DNA-binding domain (DBD) that, if used, would result in the omission of ten amino acids from the C-terminal extension of the DBD (indicated by an arrow in Figure 1A). This type of spliced mRNA isoform has been identified in *D. immitis* (C. Shea, J. Richer and C. V. Maina, unpublished results). We were unable to clone such an isoform (EcRB) from *B. malayi* by RT-PCR. However, we cannot exclude the possibility that *Bma-EcRB* is expressed in specific tissues or developmental stages not represented in the libraries or RNA used.

### Sequence and phylogenetic analysis of Bma-EcR

**Figure 2:**
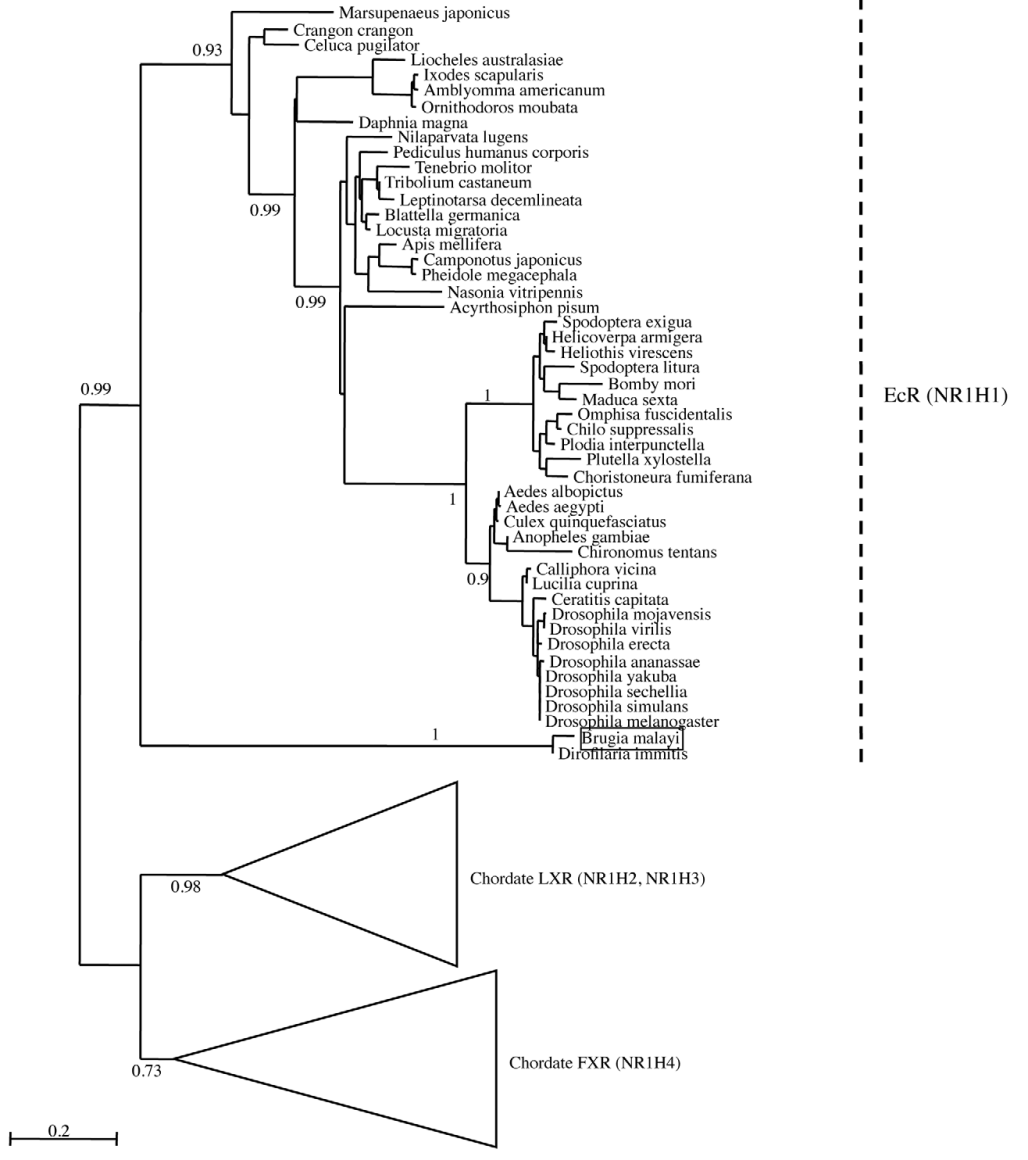
Phylogenetic analysis of Bma-EcR and related nuclear receptor sequences. Maximum Likelihood tree of EcR and related nuclear receptors (NR1H group). The alignment with gaps includes 1139 sites. Numbers at key nodes are the fraction of aLRT support out of 1000 replicates. Bma-EcR is indicated by the *Brugia malayi* label (boxed). The scale bar represents amino acid substitutions per site. Chordate and vertebrate sequences were collapsed on this figure for readability. The complete EcR tree with all branches is shown in Fig. S2.

Bma-EcR shows strongest similarity to the NR1H group of nuclear receptors typified by the insect EcRs and mammalian FXR and LXR receptors (Figs. 1B, 2). The strongest similarity is in the DBD which contains the canonical C_4_ zinc finger structure of nuclear receptors. This domain is 10 amino acids longer in Bma-EcR than in the homologous region of the other EcRs. However, as indicated above, exclusion of these 10 amino acids by alternative splicing of this site (isoform B) would result in a better alignment of Bma-EcR with the other EcRs (Fig. 1B). The LBD shows significant similarity in the regions encoding helices 3–10 [48]. An exception is found in the region of helix 11–12 (Fig. 1B). Helix 12 of insect EcRs contains the AF2 motif, responsible for ligand-dependent transcriptional activation. Although predictions of secondary structure of the *Bma-EcR* LBD protein sequence indicate helical folding of putative helices 3–10, no helical propensity is predicted in the region of helix 12 (data not shown). Immediately following the helix 12 region a glutamine-rich helical segment is present. Glutamine-rich sequences are often associated with transcription activation domains [49]. These differences make Bma-EcR an unusual member of the receptor family that perhaps uses a different mechanism for ligand-dependent activation.

Global phylogenetic analysis (see Supporting Information Fig. S1) places *Bma-EcR* with arthropod EcRs. The position of *Bma-EcR* is strongly supported (99% aLRT support) in a phylogenetic tree of the sub-family (Figures 2 and S2). The branch leading to *Bma-EcR* is long, indicating a relatively derived sequence, but not more derived than that of dipteran EcRs, for example. Separate analysis of the DBD and LBD produced similar tree topologies, especially concerning the position of *Bma-EcR*.

### BmaEcR expression analysis

**Figure 3:**
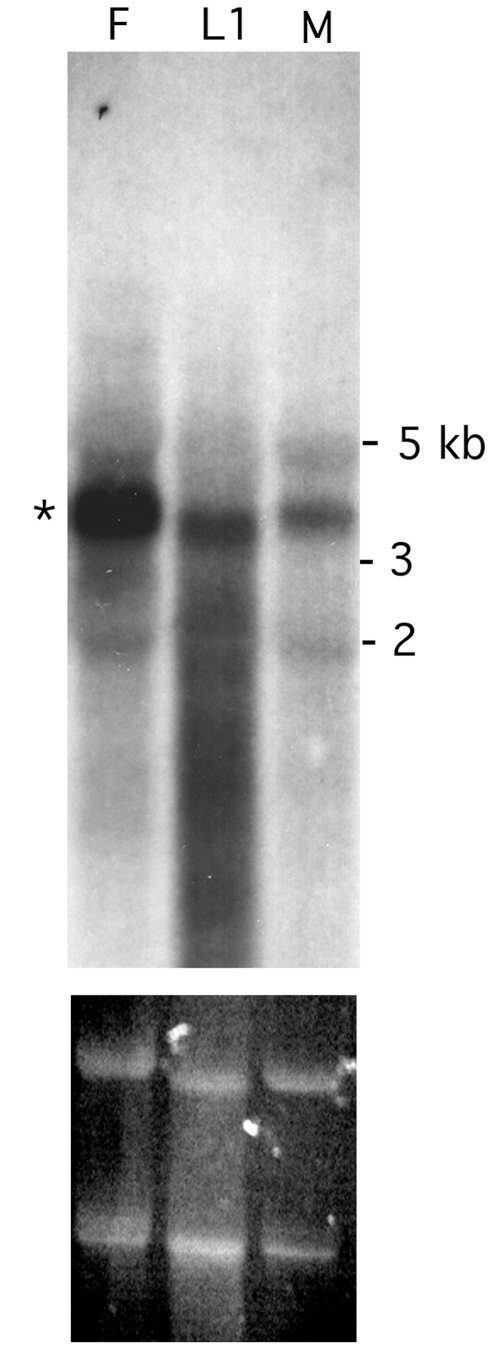
*Bma-EcR* expression in adult females, males and microfilaria. Northern blot analysis of total RNA from *B. malayi* females (F), L1 larvae (L1) and males (M) shows the presence of a predominant RNA species of approximately 3.5–4 kb (marked by an asterisk). The ethidium bromide-stained ribosomal RNA (below) reflects the amount of total RNA loaded in each lane. The apparent difference in mobility of the L1 sample is probably associated with electrophoresis rather than actual mRNA size, as it is also observed in both ribosomal RNA species detected by ethidium bromide (lower panel).

Northern blot analysis was used to establish the expression pattern of *Bma-EcR* in *Brugia* adult females, males, and L1 microfilaria. The fragment used as the probe encompassed the coding sequence common to both mRNA isoforms identified. A predominant species of approximately 3.75–4 Kb was present in all RNA samples tested (Fig. 3), which was consistent with our detection by RT-PCR of the *Bma-EcR* isoforms in libraries from those same stages, and implies the existence of longer 5′ and/or 3′ untranslated regions than are present in our cloned cDNA species. Shorter minor RNA species are detectable which may indicate the existence of additional isoforms (Fig. 3).

### Bma-EcR dimerization with RXR and USP

**Figure 4:**
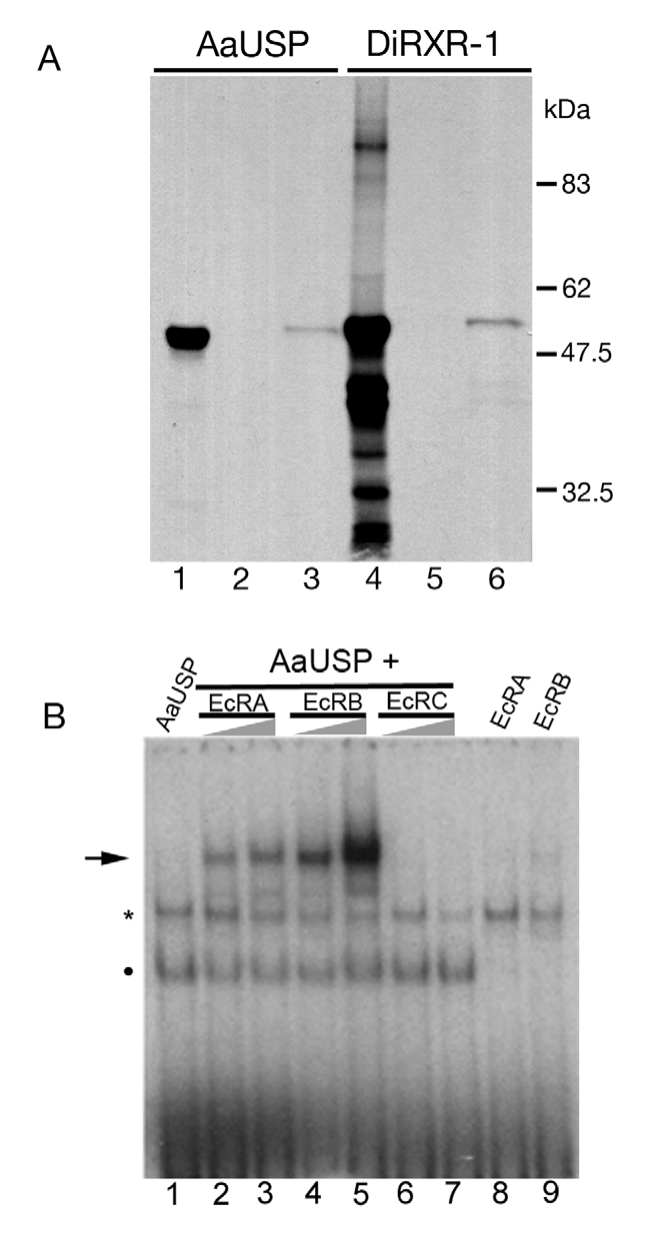
Bma-EcR heterodimerization with USP and DNA binding. (A)GST or GST:Bma-EcR fusion protein immobilized on glutathione agarose beads was incubated with ^35^S-labeled *in vitro* translated mosquito USP (AaUSP) or *D. immitis* Di-RXR-1 as indicated. After washing, the bound protein was detected by SDS-PAGE and autoradiography. Lanes 1 and 4 correspond to AaUSP and Di-RXR-1, respectively, which were used as the input in this assay. Lanes 2 and 5 correspond to beads coated with GST alone, exposed to AaUSP or Di-RXR-1, respectively. Lanes 3 and 6 correspond to AaUSP and Di-RXR-1, respectively, bound to GST:Bma-EcR. (B) Gel-shift analysis of Bma-EcR-A, putative -B, and -C isoforms, combined with AaUSP on a palindromic EcRE. Bma-EcR-A and -C heterodimerize with USP and bind the EcRE. Increasing amounts of each Bma-EcR isoform (as indicated above the triangles) were incubated with AaUSP and the DNA probe as indicated. A specific band is observed when Bma-EcR-A or -B is combined with AaUSP (arrow). Bma-EcRC shows no binding. A band corresponding to AaUSP alone is indicated by a dot. No binding was observed in the absence of AaUSP with either isoform. A non specific band from the rabbit reticulocyte lysate (asterisk) is present in all lanes. Lane 1: only USP. Lanes 2, 4 and 6: USP with 1 µL EcR. Lanes 3, 5, and 7: USP with 2 µL EcR. Lanes 8, 9 only the corresponding EcR. The higher intensity of the EcRB band is likely the result of higher concentration of this protein (see Fig. S3).

EcRs heterodimerize with Ultraspiracle (USP) proteins to form functional ecdysone receptors that bind to ecdysteroid ligands and ecdysone response elements (EcREs) [35]. In order to test whether Bma-EcR heterodimerizes with a canonical insect USP or its filarial homologue Di-RXR-1 [36], an *in vitro* binding assay was carried out. *In vitro* translated ^35^S-labeled Di-RXR-1 or *Aedes aegypti* USP (AaUSP) were incubated with GST or GST:Bma-EcR fusion proteins immobilized on glutathione-beads. Specific bands corresponding to the full length AaUSP and Di-RXR-1 were detected bound to GST:Bma-EcR (Fig. 4A). No binding to GST alone was detected with either protein bait. While the *in vitro* translation of AaUSP resulted in the production of a major protein species of the predicted full length AaUSP (Fig. 4A, lane 1), *in vitro* translation of Di-RXR-1 produced multiple protein species (Fig. 4 lane 4) including one corresponding to full-length Di-RXR-1 (ca 55 kD), which specifically bound to GST:Bma-EcR (Fig. 4A lane 6). These results indicate that Bma-EcR protein, like EcR, is capable of heterodimerization with USP protein *in vitro*.

### BmaEcR DNA binding properties

Having established that Bma-EcR can dimerize with USP/RXRs, we investigated the DNA binding properties of the two isolated protein isoforms of *Bma-EcR* (Forms A and C) to a palindromic ecdysone response element (PAL-1 EcRE) based on the *Drosophila hsp27* ecdysone response gene [43], using EMSA. In addition to the cloned *Bma-EcRA* and *Bma-EcRC* mRNA isoforms, a construct lacking the 10 amino acids downstream of the zinc finger domain was engineered (putative Bma-EcRB). The three Bma-EcR isoforms and AaUSP (as the heterodimerization partner) were produced in rabbit reticulocyte lysates and their relative amounts were estimated using ^35^S-met labeling and autoradiography (Fig S3). An equal amount of AaUSP-containing reticulocyte lysate was incubated with increasing amounts of each Bma-EcR isoform preparation and ^32^P-labeled EcRE prior to analysis by native polyacrylamide gel electrophoresis. AaUSP produces a specific band with the EcRE as has been shown before [50] (Fig. 4B, dot), which migrates faster than a nonspecific band produced by the reticulocyte lysate (Fig. 4B, asterisk). Both Bma-EcRA and -B produced an additional slower migrating band consistent with a heterodimer bound to the probe (Fig. 4B, lanes 2–5, arrow). In contrast, no additional band is detected with Bma-EcRC (Fig. 4B, lanes 6–7). This result is not unexpected given that Bma-EcRC, which contains a premature stop codon and encodes a protein with a truncated LBD, lacks essential structural features for heterodimerization. Neither Bma-EcRA nor Bma-EcRB bound substantially to the EcRE in the absence of AaUSP (Fig. 4B, lanes 8–9). This *in vitro* analysis of Bma-EcR heterodimerization with AaUSP and binding to an EcRE suggests that Bma-EcR has DNA-binding properties similar to those of ecdysone receptors.

### Cloning of a *B. malayi* RXR homolog

**Figure 5:**
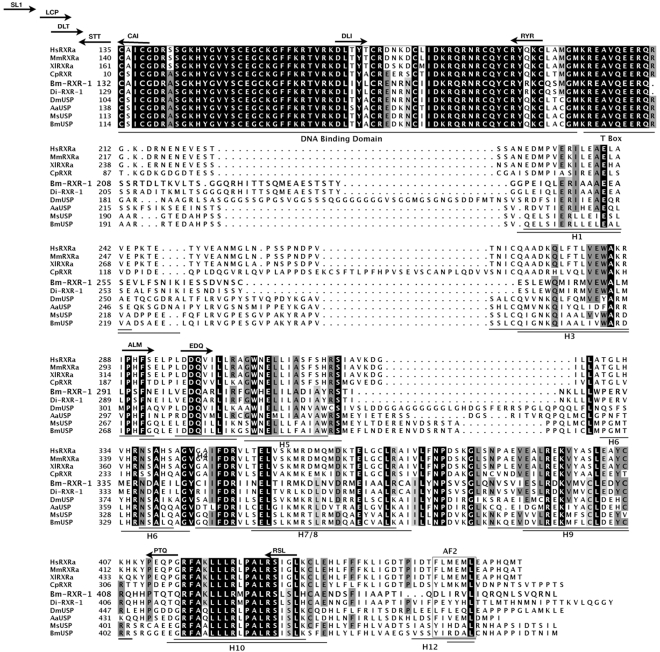
Protein sequence alignment of Bma-RXR with RXRs and USPs. RXR and USP sequences are shown above and below the filarial sequences respectively. Residues that are identical in a majority of the USP/RXRs shown are shaded in black. Residues that are biochemically similar are shadowed in gray. Functional regions (DBD, T/A Box and LBD helices) are underlined and amino acid residue numbers are indicated. Bma-RXR residues shared with all RXRs or USPs, and the AF2 are boxed. PCR primers used in cloning and mentioned in the text are indicated using the same labeling convention as in Fig. 1B.

The dimerization properties of Bma-EcR and the identification of *rxr* [36] and *EcR* homologs in the dog filarial parasite *D. immitis* pointed to the likelihood that an *rxr* homolog also exists in other filarial nematodes. Using degenerate PCR primers we were able to clone a fragment with high sequence similarity to Di-RXR-1 from *O. volvulus* cDNA (see Experimental Procedures; sequence deposited in GenBank ). In *B. malayi*, however, although we searched for an RXR type receptor in the genomic libraries available using *Di-rxr-1* as a probe, no strongly hybridizing sequences were detected. While this work was in progress genomic data from the *B. malayi* genome project became available, which provided us with an alternative route to clone the *B. malayi* RXR/USP [51]. Using the sequence information from the other filarial species as well as the *Brugia malayi* genome project we designed a combined RT-PCR and RACE approach (described in detail in Experimental Procedures) that allowed us to obtain clones for the *B. malayi* homolog of RXR which we named *Bma-RXR*. The longest cDNA sequence identified for *Bma-RXR* is 1398 bp and encodes a 465 amino acid protein that has strong similarity to *D. immitis* Di-RXR-1 (100% amino acid identity in the DBD and 83% in the LBD). The amino acid sequence similarity between the *B. malayi* and *D. immitis* RXRs substantially deteriorates in the last exon. Interestingly, the last exon corresponds to the helix 12 region of the LBD where the activation function AF-2 usually resides (Fig. 5). This LBD region is also highly dissimilar between the filarial nematode RXRs and their homologs in other non-nematode species. Notably the motif LIRVL consistent with the RXR AF2 is found in Bma-RXR but not Di-RXR-1.

### Phylogenetic analysis of Bma-RXR

**Figure 6:**
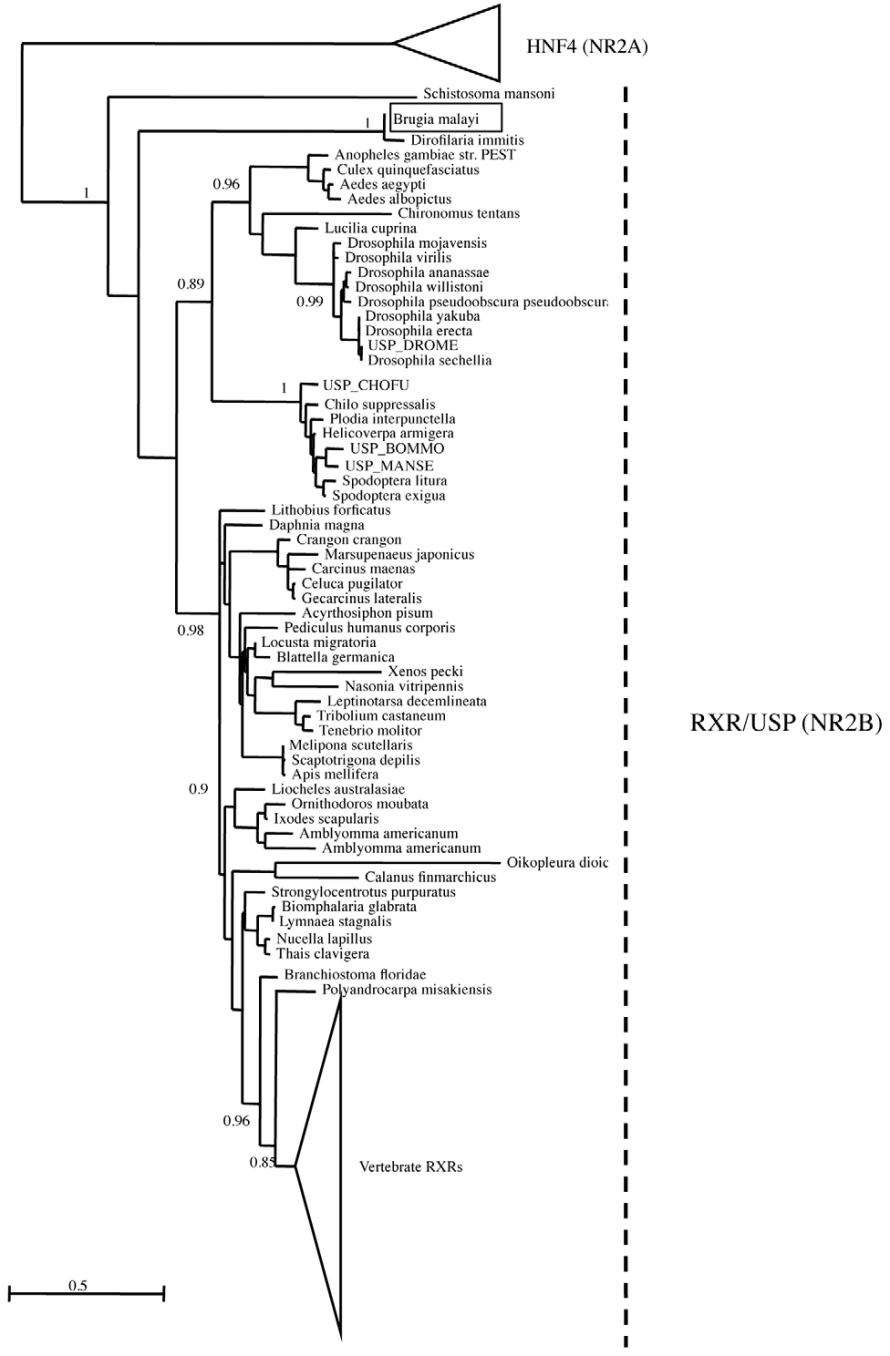
Phylogenetic analysis of *Bma-RXR* and related nuclear receptor sequences. Maximum Likelihood tree of Bma-RXR and related nuclear receptors (NR2B group). The alignment with gaps includes 514 sites. Numbers at key nodes are aLRT support out of 1000 replicates. Bma-RXR is indicated by the boxed label Brugia malayi. The scale bar represents amino acid substitutions per site. Vertebrate and outgroup sequences were collapsed on this figure for readability. The complete RXR tree with all branches is shown in Fig. S2.

Similarly to Bma-EcR, global phylogenetic analysis places Bma-RXR together with USPs and RXRs (Supplementary material Figs. S1 and S2.). Using HNF4s as the outgroup, there is 100% aALRT (approximate Likelihood Ratio Test ) support to place Bma-RXR in the USP/RXR sub-family (Fig. 6). Relationships among arthropod USP/RXRs and Bma-RXR are not well resolved (aALRTs under 50%), but Bma-RXR groups strongly with Di-RXR-1. The grouping of Bma-RXR among USP/RXRs remains the same whether the *Schistosoma mansoni* sequences are included or not in the tree (data not shown). The *Schistosoma* sequences are extremely divergent, to the extent of not being phylogenetically informative [52],[53], and branch at the base of the tree. While it is known that dipteran and lepidopteran USPs evolve especially fast [53], Di-RXR-1 and Bma-RXR appear to have evolved even faster. Separate phylogenies of the DBD and LBD (not shown) indicate that this is entirely due to a very derived LBD. This observation is consistent with the alignment. The DBD on the other hand has evolved slowly, like the DBDs of its homologs in other species.

### Bma-RXR expression

**Figure 7:**
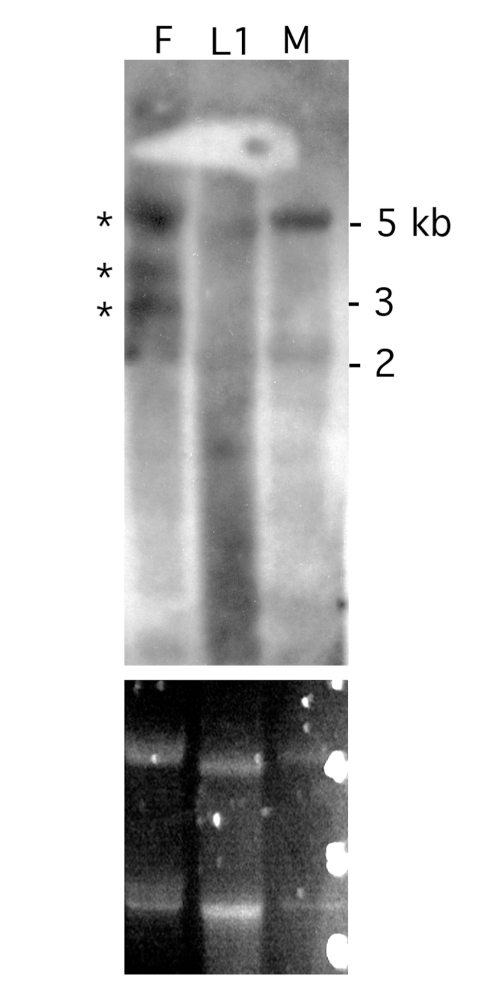
*Bma-RXR* is expressed in *B. malayi* females, males and microfilaria. Northern blot analysis of total RNA from females (F), L1 larvae (L1) and males (M) reveals 3 distinguishable RNA species of *Bma-RXR* in females (marked by asterisks). The largest of the three (around 5 Kb) is also detected in males and at low levels in L1 larvae. The ethidium bromide-stained ribosomal RNA (lower panel) reflects the amount of total RNA loaded in each lane. The apparent difference in mobility of the L1 sample is probably associated with electrophoresis as suggested by the ethidium stained gel.

Expression of *Bma-RXR* was analyzed in adult females, males, and L1 larvae by Northen blot analysis (Fig. 7). A ∼5kb RNA species was clearly detected in female and male RNA samples. Low levels of the ∼5 kb *Bma-RXR* RNA species were also observed in the L1 RNA sample. Two additional *Bma-RXR* bands of approximately 3.75 kb and 3 kb were also detected in adult females. The presence of *Bma-RXR* mRNA in males and L1 larvae was in agreement with RT-PCR results (data not shown).

### Bma-EcR heterodimerization and ligand-dependence *in vivo*

To further characterize the properties of Bma-EcR we tested whether Bma-EcR, by virtue of its LBD, is capable of forming a dimer with Bma-RXR, its putative native partner, to constitute a functional receptor and transduce the hormonal signal of ecdysteroids in a cellular context. The assay we employed takes advantage of the fact that the LBD of nuclear receptors can function in a modular fashion fused to heterologous DNA binding domains such as the GAL4 DBD [43],[44]. In order to test the ability of Bma-EcR LBD to activate transcription of a reporter gene in response to a particular hormone ligand, NIH 3T3 cells were co-transfected with GAL4:Bma-EcR(LBD) in combination with RXR LBDs fused to VP16. In addition to the Bma-RXR(LBD) we tested human HsRXR and Hs-LmRXR(LBD) (a chimeric human-locust LBD). The latter was selected because it shows no constitutive dimerization and high ligand-dependent activity when partnered with other ecdysone receptors [44] (and ). The transfected cells were tested for trans-activation in the absence or presence of either the ecdysteroid Ponasterone-A or the synthetic ecdysone agonist RSL1 by assaying luciferase activity.

**Figure 8:**
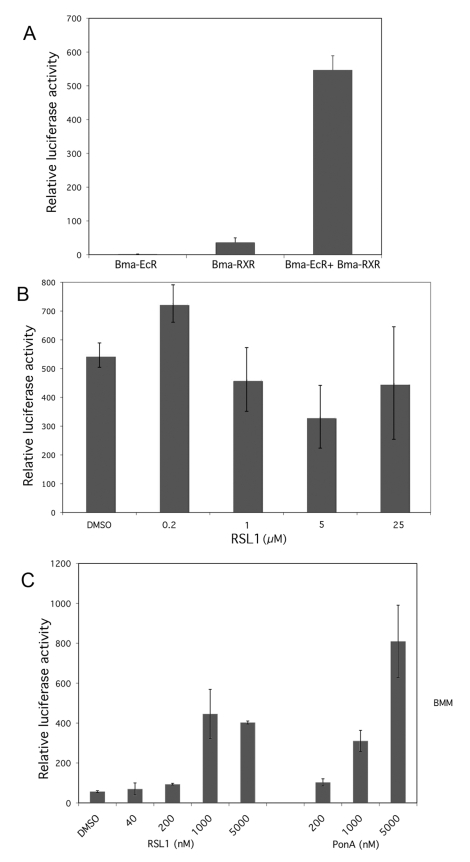
Heterodimerization of BmaEcR / BmaRXR and ligand specific responses of BmaEcR in mammalian cells. (A) Increased luciferase activity indicates constitutive heterodimerization of the Bma-EcR and Bma-RXR LBDs. The LBDs of BmaEcR and Bma-RXR were used in a two-hybrid format to generate the fusions GAL4:Bma-EcR(LBD) and VP16:Bma-RXR(LBD). These were transfected alone or together in NIH-3T3 cells. A reporter containing Luciferase downstream of the GAL4 response element was co-transfected in NIH-3T3 cells. (B) GAL4:Bma-EcR(LBD) and VP16:Bma-RXR(LBD) were transfected together as above and the cells were treated with the indicated concentrations of RSL-1 or solvent alone (DMSO). No significant activation in response to the ligand is observed. (C) Transactivation of the luciferase reporter indicates ligand dependent response of the Bma-EcR LBD. GAL4:Bma-EcR(LBD) and VP16:HsLmRXR(LBD) were transfected in NIH-3T3 cells which were treated with DMSO or increasing concentrations of RSL-1, or Ponasterone A (PonA) as indicated. HsLmRXR(LBD) is a chimeric LBD consisting of human and locust RXR LBDs. It shows no constitutive dimerization with Bma-EcR (Fig. S4) or other EcR LBDs [44].

Significant transactivation was detected when GAL4:Bma-EcR(LBD) was partnered with Bma-RXR(LBD) (Fig. 8A). The addition of RSL1 (Fig. 8B) or Ponasterone A (data not shown) had no further stimulatory effect on the detected activity. These data demonstrate that Bma-EcR and Bma-RXR are *bona fide* nuclear receptor partners and that, like their insect counterparts, they avidly dimerize in the absence of ligand. The ligands apparently cannot appreciably increase the heterodimer's ability to activate transcription above that of the VP16 activation domain in this assay.

Significant ligand-dependent transcriptional activation of luciferase was detected, however, when GAL4:Bma-EcR(LBD) was partnered with the chimeric VP16:Hs-LmRXR and treated either with Ponasterone-A or RSL1 (Fig. 8C). This is likely the result of ligand-dependent dimerization of the two receptor LBD fusions and subsequent trans-activation *via* the VP16 activation domain. This result clearly demonstrates the ability of Bma-EcR LBD to transduce the action of the ecdysteroid Ponasterone-A and the ecdysteroid agonist, RSL1 in the transfected cells.

The dimerization and transactivation studies presented here show that Bma-EcR is able to heterodimerize with Bma-RXR in a cellular context and capable of triggering a transcriptional response in an ecdysteroid-specific manner. These observations taken together along with their expression profile suggest that Bma-EcR and Bma-RXR have the prerequisite functional properties to constitute a functional *Brugia malayi* ecdysone receptor.

### Ecdysone-dependent transcription in *B. malayi*: A reporter assay

**Figure 9:**
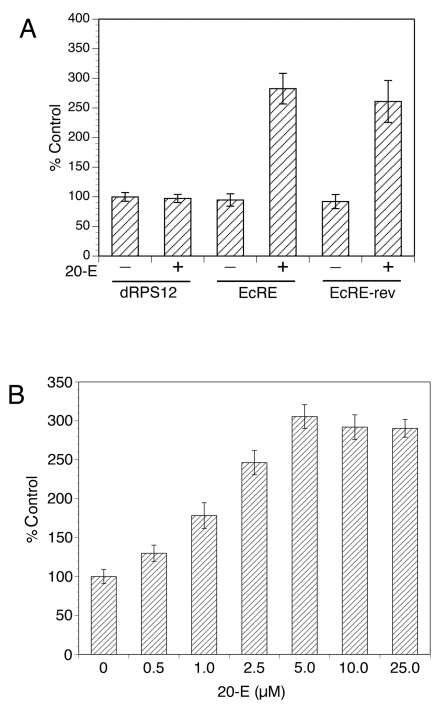
Ecdysone-dependent transcriptional activation in *B. malayi* embryos. (A) 20-E treatment causes a robust transcriptional activation with mediated by a consensus EcRE embedded in a *B. malayi* transcriptional reporter. Five copies of the EcRE used in the DNA binding assay (Fig. 4) were cloned in both orientations with respect to the direction of transcription in the BmRPS12-luciferase vector. Both reporters (EcRE and EcRE-rev) and the vector alone (dRPS12) were transformed into *B. malayi* embryos which were cultured in the presence of 20-E (+) or solvent alone (−) for 48 hrs and processed for luciferase assays. (B) 20-E causes a dose-response activation of the EcRE reporter. *B. malayi* embryos transformed with the BmRPS12- EcRE luciferase reporter were treated with the indicated concentrations of 20-OH ecdysone (in µM) or solvent alone (0). The luciferase activity obtained from solvent only treatment with the empty vector was set to 100%.

The existence of homologs for both protein components of Ecdysone Receptor in *B. malayi* which possess functional dimerization and DNA binding properties, and the earlier pharmacological observations by H. Rees [33],[34] suggest that ecdysone could function as a transcriptional regulatory ligand in *B. malayi*. To directly test this hypothesis, we employed a recently established transient transformation technique to explore whether ecdysteroids can activate transcription in *B. malayi* using a reporter assay. Recent studies have demonstrated that the 5′ UTR of the gene encoding the 12 kDa small subunit ribosomal protein of *B. malayi* (BmRPS12) was capable of acting as a promoter when used to drive the expression of a luciferase reporter gene in transiently transfected *B. malayi* embryos [45]. The BmRPS12 promoter contains 5 ¾ copies of an almost exact 44 nt repeat that acts as an enhancer element [45]. This promoter construct driving the expression of firefly luciferase (construct BmRPS12 (−641 to −1)/luc) was used to develop a reporter for *B. malayi* in which the enhancer repeat element was replaced with canonical ecdysone response elements (EcREs). We constructed the EcRE-BmRPS12-luciferase reporter (as described in Methods) using the PAL-1 element that Bma-ECR is capable of binding *in vitro* (Fig. 4B). This construct was tested for transcriptional activity in transfected *B. malayi* embryos, which were exposed to 20-OH ecdysone (20-E), or solvent alone, before being assayed for luciferase reporter activity. As shown in Fig. 9A ecdysteroid treatment resulted in a significant increase of reporter gene activity in cultures exposed to 20-E relative to control cultures (transfected in parallel with the same construct but exposed to solvent alone). This response to 20-E requires the presence of the EcRE sequence, since a construct lacking the EcRE did not exhibit any increase in luciferase activity in response to 20-E. Similarly, the response was strictly dependent on hormone, as constructs containing the EcRE produced levels of activity that were not significantly different from those obtained with the construct lacking the EcRE, in the absence of 20-E. Constructs containing the EcRE in both orientations were equally responsive to 20-E treatment, in keeping with previous studies demonstrating the symmetric nature of the binding of nuclear hormone receptors to their cognate response elements [43]. The response to the 20-OH ecdysone was dose-dependent, reaching a plateau at 5 µM (Fig. 9B). These results provide molecular evidence for the function of ecdysone in transcriptional responses of *B. malayi* and reveal the functional operation of a corresponding signaling system.

## Discussion

Molting in ecdyzosoans has been studied most extensively in insects. In insects EcR and USP initiate the transduction of the molt-triggering signal [4],[9]. Molting progression is mediated by the expression and activation of a number of well-characterized genes, including additional nuclear receptors [16],[17],[54,]. In contrast, in nematodes molting initiation and the molecular signaling responsible for its progression are only now starting to be understood. An RNAi screen in *C. elegans* for genes that are involved in molting has revealed a large number of “molting” genes, which encode proteins ranging from transcription factors and intercellular signaling molecules to proteases and protease inhibitors. However, no signal has been specifically identified as being the putative molting trigger [55]. Expression profiles of *C. elegans* “ecdysone cascade” nuclear receptors during molting cycles parallel the expression of their homologs in insects [56], and *nhr-23*, *nhr-25*, *nhr-41*, and *nhr-85*, the *C. elegans* orthologs of *DHR3*, *Ftz-F1*, *DHR78*, and *E75*, respectively, have been shown to be important for proper molting and/or dauer larva formation [26], [56]–[58]. The fact that the *C. elegans* genome contains no identifiable homologs of *EcR* or *rxr* [23] and that no ecdysteroids have been identified in this nematode, has led to the suggestion that ecdysone itself is unlikely to be the molting hormone in this free living nematode [55].

Our previous studies demonstrated the existence of an *rxr* homolog in the canine filarial nematode *D. immitis* [36]. The isolation of *Di-rxr-1* indicated that, in contrast to *C. elegans*, filarial nematodes might contain different sets of NRs. The isolation of homologs of *EcR* and *rxr* in *Brugia malayi* presented here demonstrates that filarial nematodes express both components of the ecdysone receptor and these nuclear receptors show dimerization, DNA binding, and hormone-binding characteristics similar to those of the canonical insect ecdysone receptors. Our phylogenetic analyses place the two receptors in the corresponding branches of the superfamily tree. They also indicate a rapid evolution of the LBDs. The LBDs of nematode RXRs are extremely divergent, on a similar scale to that of *Schistosoma* RXR LBD. Subsequent to our identification of *EcR* and *rxr* homologs in *Brugia*, the sequencing of the genome was completed, identifying additional putative nuclear receptors in the ecdysone signaling cascade [51].

We cloned two *Bma-EcR* and one *Bma-rxr* mRNA isoforms. Northern blot analyses revealed *Bma-EcR* and *Bma-rxr* expression in adult males, females and L1s. In addition, RT-PCR analyses indicate that *Bma-EcR* is also present in L1, L2 and L3 larval stages. Since females contain developing embryos, it is not possible to differentiate between embryonic and female-specific expression of these two nuclear receptors in *B. malayi*. In insects *EcR* has been shown to be critical for both embryonic development and oogenesis [15],[59],[60] and in filarial nematodes ecdysone treatment releases meiotic arrest and stimulates microfilaria release [34]. Expression of *EcR* and *rxr* homologs in *B. malayi* females points to possible functions of the ecdysone receptor also in nematode oogenesis and/or embryogenesis.

The expression pattern of *Bma-RXR* differs somewhat from the expression pattern of the other filarial *rxr* identified to date, *Di- rxr-1*, which is expressed in males but not females [36]. In insects the rxr homologue “*Ultraspiracle*” (USP) is considered the main functional partner of EcR and as such its expression overlaps with that of *EcR* [5]. This also seems to be the case in *B. malayi*, where we observed that at least one isoform of *Bma-rxr* has an overlapping expression pattern with *Bma-EcR*. However, two other *Bma-rxr* isoforms appear to be specifically expressed only in females.

The sequence differences of *B. malayi* and *D. immitis* RXR may mirror differences in expression patterns of the two RXR homologues. Whether these differences in sequence and expression pattern correlate with differences in ligand interaction and/or function remains an open question.

Both Bma-EcRA and a putative isoform B are able to bind a canonical ecdysone response element (EcRE) when partnered with USP. The question of whether isoform B exists in *B. malayi* (as in *D. immitis*) remains unanswered. We have shown that such an isoform is biochemically active, being able to dimerize with an insect USP and bind EcRE *in vitro*. Furthermore, isoform B is the most similar to the insect EcRs. Bma-EcRB contains a shorter (i.e. canonical) “T-box” region than Bma-EcRA (Fig. 1). The “T-box” region has been described as being able to modulate DNA binding to extended hormone response elements [61]. The presence of possible sequence variation in the “T-box” region in these two *Bma-EcR* variants could point to the possibility of differences in isoform-specific interactions with DNA target sequences.

Bma-EcRC contains a truncated LBD, and it is similar in organization to the estrogen-alpha variant Delta-5, which displays dominant-negative activity [62]. As we have shown, isoform C is unable to dimerize with a *bona fide* USP to bind the palindromic EcRE. These data suggest that Bma-EcRC may carry out a novel function that is independent of any interactions with an RXR partner. Establishing the role of *Bma-EcRC* is the aim of future investigations.

The sequence in the region of helices 11–12 in the LBD of *B. malayi* and *D. immitis* EcR and RXR homologues is strikingly divergent when compared to each other and to other EcRs and RXRs respectively. The most prominent feature in Bma-EcR is the absence of conserved helix 12 residues. This difference raises the question of what constitutes a functional activation function corresponding to AF2 in these nematode members of the nuclear receptor family. Our transcriptional activation assay results clearly show that the two receptors can dimerize and that the LBD of Bma-EcR is capable of transducing an ecdysteroid signal in a cellular context. Even though our analysis was carried out in a heterologous system, this type of assay has been shown to be highly informative for LBD-ligand interactions [44]. In this system, however, strong constitutive dimerization of receptor partners can obscure possible transcriptional effects of the ligand. Our results obtained with the chimeric RXR-LBD (which confers low constitutive dimerization) as a partner, indicate that the Bma-EcR LBD does show an ecdysteroid response. Evidence of hormone binding from these transactivation assays and the absence of a recognizable AF2 motif in Bma-EcR suggest that this receptor utilizes different features to achieve equivalent transcriptional functions than its insect counterparts.

The identification of the putative ecdysone receptor components presented here provides strong support to the long standing hypothesis that ecdysteroids play a role in filarial nematode embryogenesis and molting similar to their role in insects.[4],[32]. Ecdysteroids have been detected in a number of nematodes (reviewed by Barker and Rees, [32]). When *in vitro* cultivation of *Onchocerca volvulus* microfilaria was attained, it was observed that the addition of 20E to the culture media resulted in L1 larva progressing to the infective L3 stage [63]. This observation is consistent with the fact that after the bloodmeal, mosquitoes raise their ecdysteroid level, which correlates with the subsequent rapid molting of the ingested L1 larvae to the L2 stage. We attempted to directly demonstrate that ecdysone can act as a transcriptional trigger *in vivo* using a transient transformation reporter assay. Indeed, significant activity was observed in response to ecdysone . Our transgenic *Brugia* experiments confirm the *in vivo* functionality of both a consensus EcRE and 20-hydroxyecdysone in measurable transcriptional activity. Although we present no data to establish that the observed activation is mediated by the receptor(s) we have cloned, our results in conjunction with previous studies on this subject confirm that filarial nematodes in particular, contain and express the gene components of a functional ecdysone signaling system that is quite similar to that of other ecdysozoa. The role of this signaling system in filarial development will be the subject of further studies. Furthermore, the existence of a functional ecdysone signaling pathway in filarial nematodes does point to the possibility of using a novel approach for the development of drugs to fight filariasis based on testing of pre-existing compounds that specifically target the ecdysone pathway [64].

## Supporting Information

Figure S1 Phylogenetic tree of all Nuclear receptors. Maximum likelihood phylogenetic tree generated with all nuclear receptor sequences obtained from SwissProt and GenBank constructed as described in the Methods. The positions of Bma-EcR and Bma-RXR reported here are indicated by arrows. The accession numbers and the statistical aLRT support for the branches are indicated.
(0.23 MB TIF)

Figure S2 Sub-trees containing EcRs and RXRs. Sub-trees from the phylogeny of Figure S1 containing all EcRs (left) or all RXRs (right). The accession numbers and the statistical aLRT support for the branches are indicated.
(0.27 MB TIF)

Figure S3 In vitro translated proteins used in Figure 4. SDS-PAGE of ^35^S-labeled in vitro translated proteins used in Figure 4 showing size and relative amounts of the three Bma-EcR isoforms and AaUSP. One µL of each in vitro translated protein was analyzed by autoradiography of the dried gel.
(0.13 MB TIF)

Figure S4 HsLmRXR-VP16 (LBD) chimera activates the reporter upon RSL1 treatment only in the presence of a responsive EcR heterodimer partner. Transactivation assay with the chimeric VP16:Hs-LmRXR(LBD) used in Figure 8. The same construct was transfected along with a Gal4: CfEcR(LBD) fusion or alone in NIH-3T3 cells using the same experimental protocols as for Figure 8. Activation of the reporter is observed only upon induction with the ecdysone agonist RSL-1. CfEcR(LBD) encodes the LBD of the ecdysteroid receptor from *Choristoneura fumiferana* [44].
(0.07 MB DOC)

Table S1 List of accession numbers for all EcR and RXR sequences used in the phylogenetic analyses shown in Figures 2 and 6.
(0.07 MB DOC)
