# Supplementary figures and images for: Molecular Evidence for a Functional Ecdysone Signaling System in Brugia malayi
Source: PLoS Negl Trop Dis. 2010 Mar 9;4(3):e625. doi: 10.1371/journal.pntd.0000625 (PMC2834746; doi:10.1371/journal.pntd.0000625)

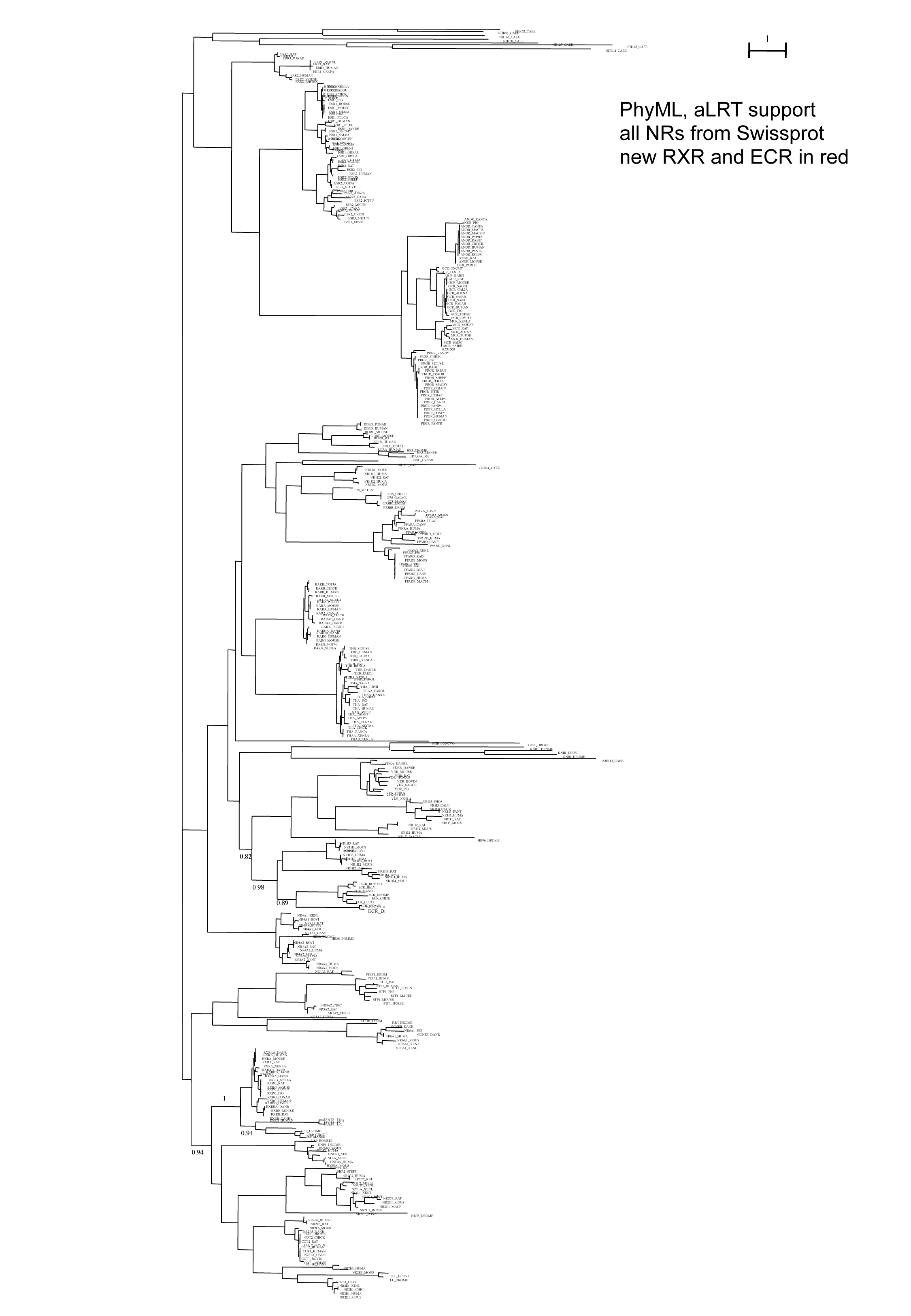

Supplement: Figure S1 — Phylogenetic tree of all Nuclear receptors. Maximum likelihood phylogenetic tree generated with all nuclear receptor sequences obtained from SwissProt and GenBank constructed as described in the Methods. The positions of Bma-EcR and Bma-RXR reported here are indicated by arrows. The accession numbers and the statistical aLRT support for the branches are indicated. (0.23 MB TIF) [file pntd.0000625.s001.tif]

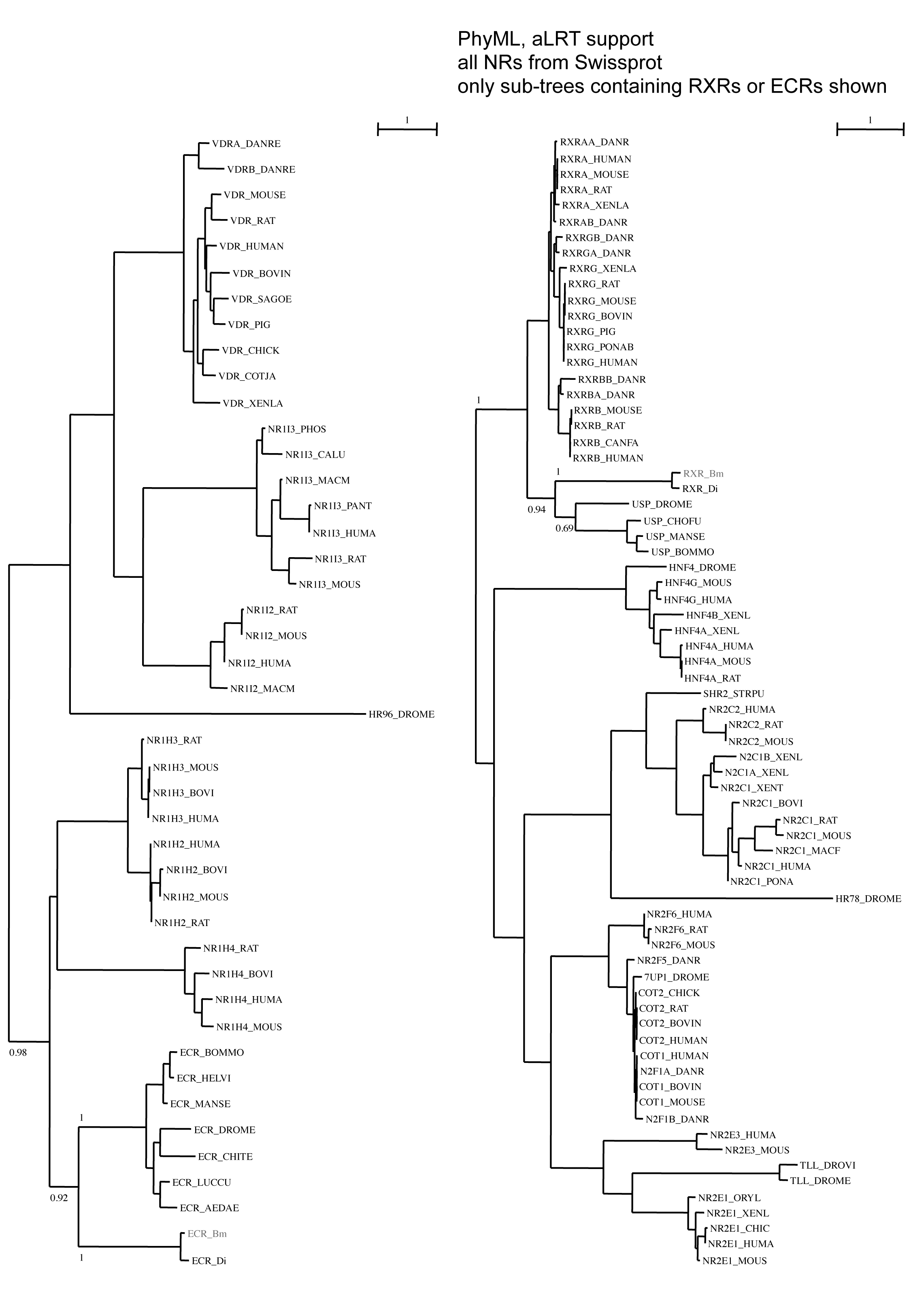

Supplement: Figure S2 — Sub-trees containing EcRs and RXRs. Sub-trees from the phylogeny of Figure S1 containing all EcRs (left) or all RXRs (right). The accession numbers and the statistical aLRT support for the branches are indicated. (0.27 MB TIF) [file pntd.0000625.s002.tif]

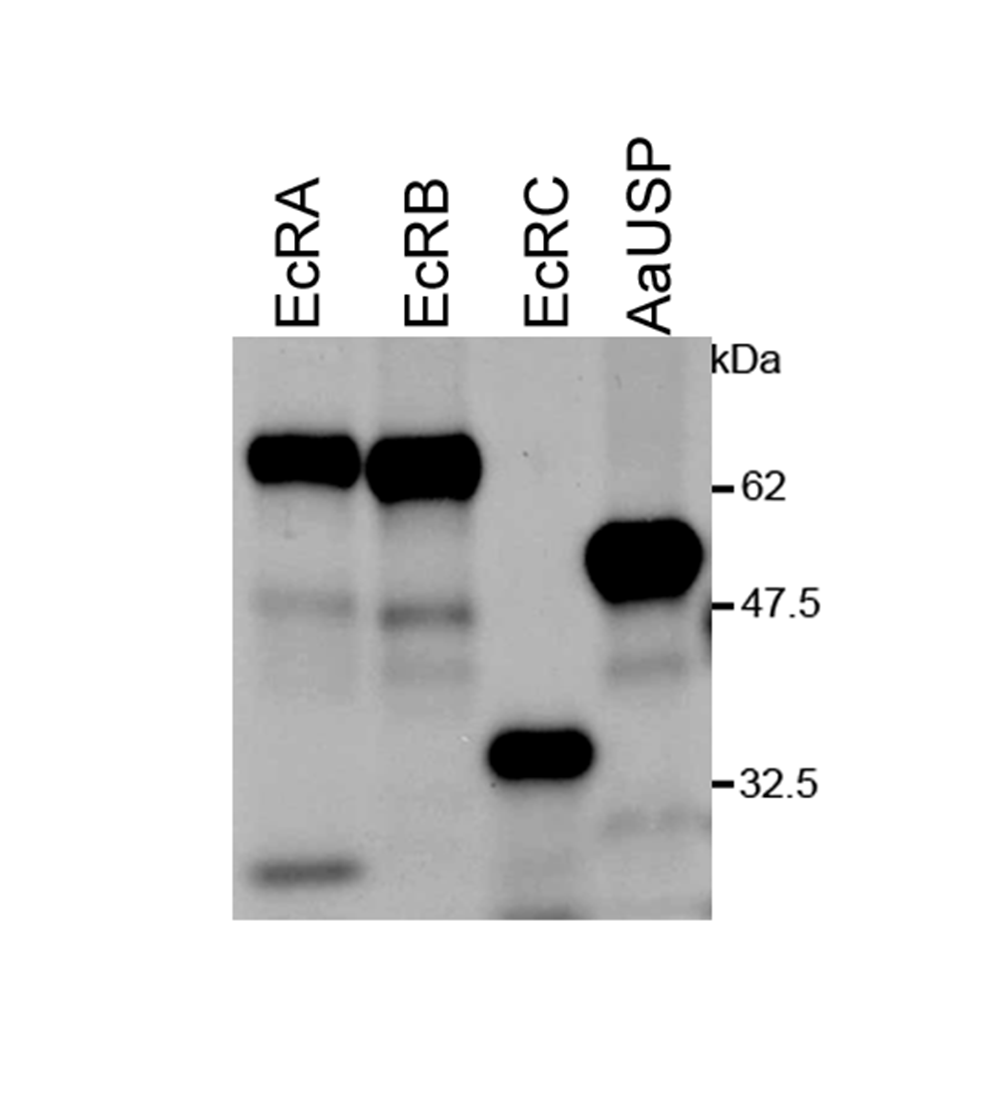

Supplement: Figure S3 — In vitro translated proteins used in Figure 4. SDS-PAGE of 35S-labeled in vitro translated proteins used in Figure 4 showing size and relative amounts of the three Bma-EcR isoforms and AaUSP. One µL of each in vitro translated protein was analyzed by autoradiography of the dried gel. (0.13 MB TIF) [file pntd.0000625.s003.tif]

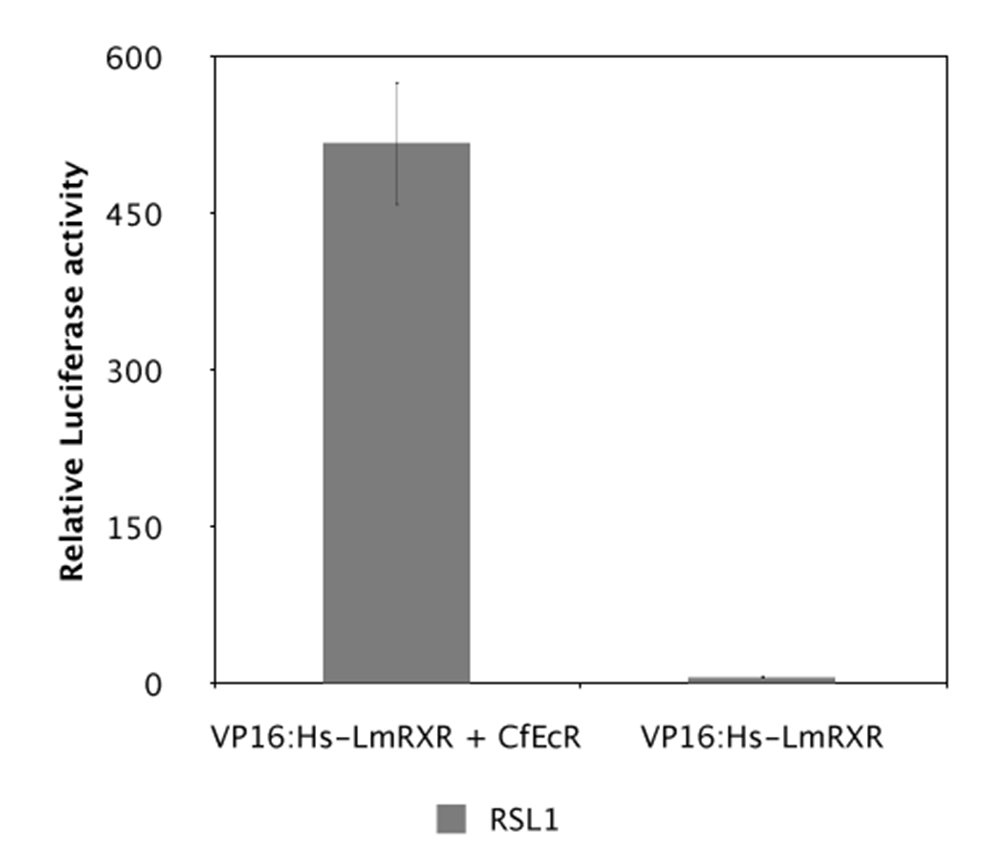

Supplement: Figure S4 — HsLmRXR-VP16 (LBD) chimera activates the reporter upon RSL1 treatment only in the presence of a responsive EcR heterodimer partner. Transactivation assay with the chimeric VP16:Hs-LmRXR(LBD) used in Figure 8. The same construct was transfected along with a Gal4: CfEcR(LBD) fusion or alone in NIH-3T3 cells using the same experimental protocols as for Figure 8. Activation of the reporter is observed only upon induction with the ecdysone agonist RSL-1. CfEcR(LBD) encodes the LBD of the ecdysteroid receptor from Choristoneura fumiferana [44]. (0.07 MB DOC) [file pntd.0000625.s004.tif]
