# Supplementary material for: Molecular Evidence for a Functional Ecdysone Signaling System in Brugia malayi
Source: PLoS Negl Trop Dis. 2010 Mar 9;4(3):e625. doi: 10.1371/journal.pntd.0000625 (PMC2834746; doi:10.1371/journal.pntd.0000625)
Supplement: Table S1 — List of accession numbers for all EcR and RXR sequences used in the phylogenetic analyses shown in Figures 2 and 6. (0.07 MB DOC) [file pntd.0000625.s005.doc]

| **EcRs** | **Accession #** |  | **RXRs** | **Accession #** |
| --- | --- | --- | --- | --- |
|  |  |  |  |  |
| *Marsupenaeus japonicus* | gi154936860 |  | *Schistosoma mansoni* | gi256089112 |
| *Crangon crangon* | gi226316403 |  | *Dirofilaria immitis* | gi19919404 |
| *Celuca pugilator* | gi13677226 |  | *Anopheles gambiae str. PEST* | gi158301222 |
| *Liocheles australasiae* | gi158302670 |  | *Culex quinquefasciatus* | gi170061655 |
| *Ixodes scapularis* | gi241997792 |  | *Aedes aegypti* | gi10946383 |
| *Amblyomma americanum* | gi2738906 |  | *Aedes albopictus* | gi6601567 |
| *Ornithodoros moubata* | gi76880161 |  | *Chironomus tentans* | gi2895868 |
| *Daphnia magna* | gi131665112 |  | *Lucilia cuprina* | gi14624971 |
| *Nilaparvata lugens* | gi226432213 |  | *Drosophila mojavensis* | gi195130249 |
| *Pediculus humanus corporis* | gi242019559 |  | *Drosophila virilis* | gi195397487 |
| *Tenebrio molitor* | gi2155008 |  | *Drosophila ananassae* | gi194768727 |
| *Tribolium castaneum* | gi166795273 |  | *Drosophila willistoni* | gi195447730 |
| *Leptinotarsa decemlineata* | gi66864090 |  | *Drosophila pseudoobscura* | gi198470596 |
| *Blattella germanica* | gi86439690 |  | *Drosophila yakuba* | gi195477854 |
| *Locusta migratoria* | gi4405799 |  | *Drosophila erecta* | gi194912875 |
| *Apis mellifera* | gi124484522 |  | *Drosophila sechellia* | gi195347797 |
| *Camponotus japonicus* | gi157041191 |  | *Chilo suppressalis* | gi27372315 |
| *Pheidole megacephala* | gi78675507 |  | *Plodia interpunctella* | gi48475404 |
| *Nasonia vitripennis* | gi226823246 |  | *Helicoverpa armigera* | gi189031270 |
| *Acyrthosiphon pisum* | gi226823254 |  | *Spodoptera litura* | gi161769557 |
| *Spodoptera exigua* | gi168812206 |  | *Spodoptera exigua* | gi187940918 |
| *Helicoverpa armigera* | gi189031268 |  | *Lithobius forficatus* | gi28628006 |
| *Heliothis virescens* | ECR HELVI |  | *Daphnia magna* | gi105872963 |
| *Spodoptera litura* | gi161769555 |  | *Crangon crangon* | gi226316409 |
| *Bomby mori* | ECR BOMMO |  | *Marsupenaeus japonicus* | gi154936862 |
| *Maduca sexta* | ECR MANSE |  | *Carcinus maenas* | gi195984199 |
| *Omphisa fuscidentalis* | gi151327629 |  | *Celuca pugilator* | gi68342537 |
| *Chilo suppressalis* | gi22761812 |  | *Gecarcinus lateralis* | gi71040954 |
| *Plodia interpunctella* | gi40362586 |  | *Acyrthosiphon pisum* | gi239735516 |
| *Plutella xylostella* | gi148515004 |  | *Pediculus humanus corporis* | gi242008309 |
| *Choristoneura fumiferana* | gi5713322 |  | *Locusta migratoria* | gi33943178 |
| *Aedes albopictus* | gi6601565 |  | *Blattella germanica* | gi70907501 |
| *Aedes aegypti* | ECR AEDAE |  | *Xenos pecki* | gi60892853 |
| *Culex quinquefasciatus* | gi170033431 |  | *Nasonia vitripennis* | gi156551053 |
| *Anopheles gambiae* | gi158300393 |  | *Leptinotarsa decemlineata* | gi66864094 |
| *Chironomus tentans* | ECR CHITE |  | *Tribolium castaneum* | gi166796104 |
| *Calliphora vicina* | gi12034940 |  | *Tenebrio molitor* | gi6983809 |
| *Lucilia cuprina* | ECR LUCCU |  | *Melipona scutellaris* | gi56608614 |
| *Ceratitis capitata* | gi3393034 |  | *Scaptotrigona depilis* | gi77632652 |
| *Drosophila mojavensis* | gi195124730 |  | *Apis mellifera* | gi58585210 |
| *Drosophila virilis* | gi195380874 |  | *Liocheles australasiae* | gi158302672 |
| *Drosophila erecta* | gi194864116 |  | *Ornithodoros moubata* | gi158962474 |
| *Drosophila ananassae* | gi194758180 |  | *Ixodes scapularis* | gi242000854 |
| *Drosophila yakuba* | gi195475632 |  | *Amblyomma americanum* | gi3098334 |
| *Drosophila sechellia* | gi195353814 |  | *Amblyomma americanum* | gi3098336 |
| *Drosophila simulans* | gi195580898 |  | *Oikopleura dioica* | gi112820307 |
| *Drosophila melanogaster* | ECR DROME |  | *Calanus finmarchicus* | gi227150229 |
|  |  |  | *Strongylocentrotus purpuratus* | gi115645626 |
|  |  |  | *Biomphalaria glabrata* | gi19386469 |
|  |  |  | *Lymnaea stagnalis* | gi57164664 |
|  |  |  | *Nucella lapillus* | gi154183751 |
|  |  |  | *Thais clavigera* | gi51873224 |
|  |  |  | *Branchiostoma floridae* | gi219478516 |
|  |  |  | *Polyandrocarpa misakiensis* | gi5631312 |
